# Chronic Heart Failure: Clinical Implications of Iron Homeostasis Disturbances Revisited

**DOI:** 10.7759/cureus.21224

**Published:** 2022-01-13

**Authors:** Leonardo P Suciadi, Joshua Henrina, Iwan Cahyo Santosa Putra, Irvan Cahyadi, Hoo Felicia Hadi Gunawan

**Affiliations:** 1 Cardiology, Siloam Hospitals Kebon Jeruk/Siloam Heart Institute, Jakarta, IDN; 2 Family Medicine, Balaraja Public Health Center, Tangerang, IDN; 3 Research, Siloam Heart Institute, Jakarta, IDN

**Keywords:** oral, intravenous, iron therapy, chronic heart failure, iron deficiency

## Abstract

Iron deficiency is prevalent in chronic heart failure (CHF) patients. Nonetheless, the diagnosis is often overlooked and, often, the treatment is commenced just when overt anemia has ensued. Therefore, a better appreciation of this disease is needed, and all seasoned cardiologists should know how to approach CHF patients with iron deficiency correctly, as mandated by clinical practice guidelines. In this comprehensive review, we describe iron homeostasis, the pathophysiologic changes of iron homeostasis, and the clinical implications of iron deficiency on CHF patients. In addition, we delineate the evolution of clinical trials, ranging from the inception to the ongoing clinical trials of iron deficiency treatment in CHF patients. Iron deficiency contributes to the worse clinical outcome of the patients. Numerous studies have reported the clinical benefit of iron supplementation, particularly in intravenous preparation, in heart failure patients regarding symptoms, functional capacity, and quality of life (QoL) improvement. Therefore, the current guidelines recommend routine screening of iron status in all newly diagnosed heart failure patients. Eventually, intravenous iron replacement is recommended for symptomatic heart failure patients with iron deficiency, irrespective of anemia.

## Introduction and background

Heart failure is one of the most important cardiovascular diseases owing to its significant impact on mortality, morbidity, hospitalization rates, and quality of life (QoL) [1]. Despite advancements regarding therapy in the fields of cardiovascular medicine in the past decade, the mortality and morbidity due to heart failure remain high. Generally, the prognosis of chronic heart failure (CHF) patients is dismal, and in comparison to other diseases, such as colorectal, breast, and prostatic cancer, its mortality is higher [2]. According to the Atherosclerosis Risk in Communities Cohort Study, mortality rates attributed to heart failure at 30 days, one year, and five years post-hospitalization were 10.4%, 22%, and 42.3%, respectively [3].

Heart failure is a clinical syndrome caused by structural abnormality and/or ventricles systolic or diastolic dysfunction, which renders the inability of heart tissues to meet and maintain metabolic demands due to inadequate systemic and peripheral oxygenation [4]. Presently, it is known that heart failure is a progressive disease caused by complex systemic mechanisms that include substrate abnormality of the myocardium, maladaptive compensation, and amplification [2,5]. Furthermore, sympathetic nerve activation and catecholamine release, renin-angiotensin-aldosterone system (RAAS), and inflammatory cytokines also play essential roles in the heart failure mechanisms [5-6].

Consequently, other than causing myocardial damage and injury, these maladaptive systemic responses inflict damage and disturbances to the vascular endothelium, kidney, skeletal muscles, bone marrow, lungs, and liver. These systemic pathomechanisms will manifest clinically as heart failure syndromes with their respective potential comorbidities in the course of illness. Various comorbidities and complications that accompany CHF will ultimately influence the progressivity, clinical symptoms, and prognosis of heart failure [4,7].

Iron deficiency is one of the comorbidities worthy of recognition in CHF patients and holds a promising and potential treatment target [8]. However, its diagnosis is often overlooked because of nonspecific findings on clinical examination and only to be explored once it culminates with anemia. Therefore, laboratory workup is needed to assess several parameters of body iron status to establish the diagnosis of iron deficiency [9]. Routine screening for iron deficiency in CHF patients is endorsed in major clinical practice guidelines for cardiologists [4,10].

Iron homeostasis derangement is a common event in chronic diseases, especially CHF [11]. Several pieces of research have shown that iron deficiency is prevalent in this disease, even without overt anemia. A cohort involving 1506 CHF subjects reported that the prevalence of iron deficiency is 50%, and, astoundingly, 45.6% of them are not anemic [12]. The predisposing factors and major predictors of iron deficiency in CHF patients are not fully elucidated, particularly in the non-anemic population. This issue is compounded by the scarcity of clinical research data or studies that included only a small number of subjects that show the correlation of several iron deficiency parameters and CHF [8].

Another controversy is whether low serum iron is a consequence of protracted CHF or a comorbid factor that influences CHFs progressivity. Moreover, the diagnostic utility of serum iron monitoring in non-anemic CHF populations is not extensively studied and rarely checked in clinical practice.

## Review

Cardiomyocyte’s metabolism and energetics in chronic heart failure

The process of energy metabolism in cardiomyocytes proceeds in three stages, i.e., substrate uptake and utilization, energy generation through oxidative phosphorylation, and energy transfer through creatinine kinase. Any derangement of these steps will ultimately cause contractility dysfunction. In CHF, several principal components needed for cardiomyocytes' energy metabolism are downregulated [13]. A study of cardiomyocyte energetics in advanced heart failure patients showed that the total concentrations of adenosine triphosphate (ATP)/adenosine diphosphate (ADP)/adenosine monophosphate (AMP), creatinine kinase activity, creatine phosphate, creatine phosphokinase, and creatine phosphate/ATP ratio were decreased [14]. Nevertheless, these alterations are not clearly distinguished whether they were biomarkers or the cause of progressivity of left ventricular dysfunction in heart failure.

In a physiologic condition, the primary substrate for cardiomyocyte's energy metabolism is the oxidation of fatty acids in the mitochondria [15]. The involved gene in this energy utilization pathway is regulated primarily by fatty acid-activated peroxisome proliferator-activated receptors (PPARs) and the PPAR-gamma coactivator-1α (PGD-1α). In an experimental animal model of heart failure, energy utilization via fatty acid oxidation was decreased due to the downregulation of genes that regulates fatty acid metabolism. Thus, in heart failure, there is a shift in substrate utilization by cardiomyocytes to glucose and energy generations via glycolysis, which decreases ATP production [13-14]. Therefore, modulation of intracellular energy metabolism is a novel and promising target in the treatment of heart failure [16].

Other causes of diminished cardiomyocyte ATP production are the abnormality of mitochondrial structures and functions. Evidence points out that in dilated cardiomyopathy, hibernating myocardium, and advanced heart failure, the mitochondria were smaller and fragmented. Furthermore, mitochondria in failing heart cardiomyocytes experienced an imbalance in dynamics between mitochondrial fusion and division [17]. The reduction of mitochondrial fusion will lead to reduced oxygen consumption and impairment of energy metabolism in the cardiomyocytes of a failing heart. In addition, it also contributes to cell death via apoptosis and/or mitophagy [17-18].

Several studies have shown that mitochondrial dysfunction played a prominent role in the progressivity of heart failure [19]. Therefore, a novel approach of mitochondrial biogenesis stimulation to increase intracellular mitochondrial concentration and the elimination or inhibition of reactive oxygen species (ROS) and to maintain mitochondrial iron homeostasis is a promising and potential therapeutic for CHF. Hopefully, it will improve cellular energy metabolism and augment myocardial contractility performance [19].

Iron metabolism and regulation

Iron is an essential micronutrient and plays a multitude of functions in all of the human cells, which are metabolic and cellular proliferation, hemoglobin synthesis, DNA synthesis, oxygen transport and reserve, a cofactor of oxidative reaction, and cellular electron transfer. Moreover, iron plays a crucial part in a myriad of cellular enzymes, including the cytochrome system in mitochondria [20]. On the other hand, unbound iron is poisonous and harmful for human tissues because it can precipitate a chemical reaction that produces reactive oxygen species such as singlet oxygen and hydroxyl radical (OH-) [21]. Fortunately, the human body is equipped with a strict iron homeostasis regulatory mechanism that simultaneously assists cellular physiology and prevents unnecessary toxicities [22]. 

The total iron in the human body is approximately 3-4 grams, and it varies between genders and ages. Erythrocytes and turnover products from erythrocytes destruction constitute two-thirds of the total body iron, and the rest is stored in the form of ferritin/hemosiderin. Therefore, erythrocytes dominate the total human body iron in the form of hemoglobin (Table 1) [23]. Approximately, only 1-2 mg of absorbed iron from the gastrointestinal tract entered the circulation [11,23]. Excessive or insufficient amounts of iron in the human body will interfere with the normal functions of the human organs.

**Table 1:** Iron distribution in the human body Source: [13] Mg = Milligrams, Kg = Kilograms

	Iron content (mg) adult man, 80 kg	Iron content (mg) adult Woman, 60 kg
Hemoglobin	2500	1700
Myoglobin/enzyme	500	300
Transferrin	3	3
Iron reserve	600-1000	0-300

Iron metabolism in the human body is a semi-closed system that is strictly regulated, and it is designed to preserve iron concentrations via recycling so that it is available for another metabolic process [24]. The human body does not own a physiologic excretion system for iron. Therefore, the pathways of iron loss are through active bleeding and the skin, gastrointestinal, and urinary tract epithelial degeneration. Repeated transfusions or iron supplementation in the long haul will potentially cause progressive iron accumulation [25].

Every stage of iron metabolism involves various molecules at the cellular level, i.e., gastrointestinal system iron absorption, bone marrow iron uptakes, reusing iron from the destruction of erythrocytes, iron storage, particularly in the liver, and its systemic regulation [24]. Iron conventionally enters the human body through the gastrointestinal absorption of digested foods. Blood transfusion and iron supplementation also increase the human body's iron content. However, the threshold between the amount of iron capably absorbed through the gastrointestinal system and the total body iron demand is narrow, more notably in the pediatric population, which is in their growth phase and in pregnant women [26]. This condition accounts for the high prevalence of iron deficiency in these populations throughout the world.

Iron absorption primarily occurs in the duodenum and the proximal part of the small intestines through the active process of intestinal villi, especially the luminal cells [11,23]. With the aid of gastric acid, the luminal cells' iron uptake from ingested foods will be optimized. Based on the iron content of foods, it can be divided into two groups, which are heme and non-heme iron. Red meat is abundant in heme iron, and it will get absorbed through the heme carrier protein (HCP) to the enterocytes, which subsequently will be degraded by heme oxygenase-1 (HO-1) [11,23]. Conversely, the non-heme iron, abundant in vegetables and fruits, is in the form of inorganic iron (Fe3+) [11,23].

The ferrireductase enzyme on enterocytes brush border reduces Fe3+ to Fe2+. Then, via the divalent metal transporter 1, Fe2+ will be transported through the cell membrane [11,23]. Some of the absorbed iron will be stored as ferritin in enterocytes while the rest of it will be transported into the circulation via ferroportin. The function of ferroportin is negatively regulated by hepcidin, the primary regulatory hormone of iron homeostasis [22,27]. The iron that enters the circulation in the form of Fe2+ will be oxidized by hephaestin into Fe3+, which is the compatible form that will bind with transferrin, a primary iron transport protein [27].

It is important to note that iron absorption is a dynamic process that is influenced by several physiologic conditions [23]. In hypoproliferative and iron deficiency anemia patients, the hepcidin serum levels are low. These conditions permit higher gastrointestinal iron absorption. On the contrary, in an excess body iron state (eg, excessive iron supplementation), hepcidin will decrease the gastrointestinal iron absorptions ability [11,22,28-29]. Iron bioavailability is also influenced by the types of iron ingested. In an iron-deficient state, heme absorption increases to 20%, whereas non-heme iron only increases to 5-10% [30]. Furthermore, a vegetarian diet also has relatively high phytate and phosphate concentrations, such as in cereal, fiber, and wheat, which decreases iron absorption to up to 50% [27]. In addition, food and beverages that are high in polyphenols, such as tea, coffee, wine, and nuts, will decrease food's iron absorption.

Apart from diet, another source of iron is recycled heme iron derived from erythrocytes breakdown. Each millimeter of blood contains 0.5 mg of iron. The average erythrocyte's life span is about 120 days, and subsequently, they got destroyed by macrophages in the reticuloendothelial system. The heme catabolism of degraded erythrocytes by HO-1 will generate free irons, which subsequently return into the circulation through ferroportin and bind with transferrin, to be used for another erythropoiesis [24]. In patients with thalassemia, iron buildup occurred owing to repeated blood transfusions. Consequently, accumulated irons will get into the circulation, and most of them are not bound by transferrin. These unbound irons are poisonous and potentially disturb normal bodily functions [25].

It has been previously mentioned that the circulating iron binds with a glycoprotein called transferrin. The majority of iron-transferrin complexes will get into the bone marrow and will be utilized for erythropoiesis [31]. The turnover rates of these complexes depend on the plasma iron level and the bone marrow erythropoiesis activity. The average clearance half-life of these complexes was 60-90 minutes, although the half-life will be shortened with an increased erythropoiesis rate and in the setting of iron deficiency [31].

Moreover, circulating iron-transferrin complexes will interact with specific transferrin receptors [24,32]. To date, two types of transferrin receptors have been discovered, which are transferrin receptor 1 (TfR1) and TfR2. These receptors are ubiquitously found in the human cells, including cardiomyocytes and hepatocytes, but the majority are found on the surface of erythroblasts in the bone marrow. TfR1 is a functional transferrin receptor in erythroblasts and is involved in erythropoiesis, whereas the TfR2 is abundant on hepatocyte surfaces with unknown iron uptake functions [33].

The interaction between iron-transferrin complexes and their corresponding receptors will cause endocytosis and internalization of the complexes within endosomes. In erythroblasts, the iron component will subsequently be utilized for heme synthesis, whereas transferrin will be returned into the circulation [24,33]. Afterward, the synthesized iron in the form of hemoglobin will be returned into the circulation as erythrocytes. The excess iron not needed for erythropoiesis will bind with apoferritin, forming ferritin, which is in charge of cellular iron storage, and some of the iron integrate as an enzyme component responsible for cellular metabolism [24,27]. The liver is the primary organ of iron storage, and excess iron is stored in the form of ferritin and hemosiderin [24].

Hepcidin, a peptide that regulates systemic iron metabolism, is a negative regulator that restricts gastrointestinal iron absorption and prevents its release from the reticuloendothelial system [23-24,28-29]. In a state of iron excess, hepcidin synthesis will be increased, and because it is an acute-phase protein, its level will rise in an inflammatory state. On the other hand, the genetic and expression abnormality of hepcidin is attributed to iron deposition in the tissues or hemochromatosis in the state of excess iron [34].

The role of myoglobin in cellular energy metabolism

Myoglobin is a cytoplasmic hemoprotein consisting of a single polypeptide of 154 amino acids. It is structurally and functionally similar to hemoglobin. The concentration of myoglobin varies among species and tissue. Myoglobin, as a cellular component in the human body, is abundantly found within the cardiomyocyte and oxidative skeletal muscle fiber [35].

Myoglobin has an essential role in cardiac and skeletal muscle physiological function. It plays an important role at the cellular level, especially in oxidative metabolism and to bind, transport, and store oxygen intracellularly [36]. Heme residue, which consists of a complex of porphyrin ring and iron ion, is a vital component of myoglobin [37]. Thus, iron is a necessary component for myoglobin function. Furthermore, the intracellular concentration of myoglobin correlates with oxidative enzyme activity, capillary density, and mitochondrial density [35]. The concentration of intracellular myoglobin can increase along with the increase of physiologic demand and ischemic conditions.

Myoglobin is the most critical protein component of muscle to keep oxygen storage (oxymyoglobin/MbO2). Myoglobin also plays a role as intracellular partial oxygen pressure (PO2) through its capacity as an oxygen reservoir and transport in tissue. This buffer function allows intracellular concentration to remain reasonably constant and homogeneous despite an increase in muscle activity, permitting an optimal oxidative metabolism within mitochondria [37]. The myoglobin function of the PO2 buffer is achieved through a mechanism of PO2 gradient regulation and oxygen transport from the sarcolemma to the mitochondria.

Myoglobin participates in facilitating oxygen diffusion to mitochondria, thus increasing oxygen influx to mitochondria, other than through a simple diffusion process [35-36]. The outer membrane of mitochondria has an unusually low resistance towards oxygen flow. Therefore, only a low pressure of oxygen is necessary for oxygen to flow towards mitochondria. Oxygen pressure around the outer membrane of mitochondria and the aid of myoglobin in oxygen diffusion ensure the availability of oxygen to oxidase cytochrome in normal cardiac conditions [38].

Another study reported the function of myoglobin as a cellular antioxidant through its ability to bind to nitric oxide (NO), a molecule with both an advantageous and a disadvantageous cellular effect [39]. NO can inhibit cytochrome C oxidase, disrupting mitochondrial respiration. It has a vasodilatory effect in cardiac blood vessels and a depressant effect in myocardial contractility. Myoglobin binds to NO through two different reactions, a direct interaction of MbO2 with NO, which then forms methemoglobinemia, and a nitrosylation of myoglobin deoxygenase (Mb), which then forms an intermediate compound of MbNO before reacting further with oxygen [40]. This clearance mechanism of NO will attenuate the bioactivity of intracellular NO, particularly in the myocardium. Myoglobin also has a peroxidase activity, supporting the role of myoglobin as an antioxidant [37].

A study on mice found a shift of myocardial metabolism from fatty acid to glucose in myoglobin-deficient mice. This shift in metabolism happens as a compensatory mechanism, as myoglobin plays a part in the binding and facilitation of fatty acid diffusion through sarcoplasm [35]. Other animal studies found significant myocardial dysfunction in a low cellular MbO2 concentration [35-36]. In myoglobin deficient mice, even when sarcomere structure and mitochondrial content are normal, there were lower cellular and molecular adaptive response towards relative hypoxia. Consequently, this condition inevitably leads to myocardial contractility dysfunction with subsequent left ventricular failure [41].

Pathomechanism of iron homeostasis in chronic heart failure

Disruption of iron homeostasis is often found in various chronic diseases with systemic manifestation, such as connective tissue disease, inflammatory bowel disease, chronic kidney disease, including CHF [42]. Furthermore, the cause is multifactorial and causes both absolute and functional iron deficiency. Mesenteric congestion and hypoperfusion are common manifestations of heart failure, resulting in anorexia and low nutritional intake, edema, and intestinal cell dysfunction. These cause an exaggerated intestinal villi degeneration that culminates with loss of iron through the gastrointestinal tract [32,43]. This condition ultimately ends with an absolute iron deficiency in heart failure patients, marked by a decrease in circulating and reserved iron [43]. Moreover, the increased pro-inflammatory cytokines in CHF patients disturb iron regulation and homeostasis, resulting in functional iron deficiency [37].

In an exaggerated inflammatory state, the concentrations of hepcidin will rise. This acute-phase protein is solely responsible for functional iron deficiency by reducing ferroportin expression, limiting gastrointestinal tract iron absorption, iron redistribution from circulation to tissue iron reservoir, and iron retention within the reticuloendothelial system [33]. Numerous inflammatory cytokines, such as tumor necrosis factor-α (TNF-α) and interferon-γ (IFN-γ), and other acute-phase reactants also play a part in an iron regulation disturbance in chronic inflammation [44].

In the early stage of CHF, circulating iron decreased by 15%-30%. In comparison, the tissue iron reservoir or ferritin can increase by 24% [43]. This situation reflects on iron dysregulation and functional iron deficiency in CHF. Moreover, as heart failure progressed, the iron reserve will concurrently decrease, signifying absolute iron deficiency [45].

A low circulating iron level consequently disrupts active erythropoiesis in bone marrow, causing anemia. Moreover, iron deficiency exclusively can also cause organ dysfunction without full-blown anemia. A previous study found that cardiomyocyte's iron content is decreased in CHF patients because of neurohormonal activation, which reduces mRNA expression, downregulates Tfr1, and ultimately prevents iron uptake [46]. Low cardiomyocyte iron levels result in lower myoglobin concentration, which functions as a tissue iron reservoir and a facilitator in cellular energy metabolism [46]. This condition has the potential to further cause dysfunction in mitochondria and cellular energy metabolism.

**Figure 1:**
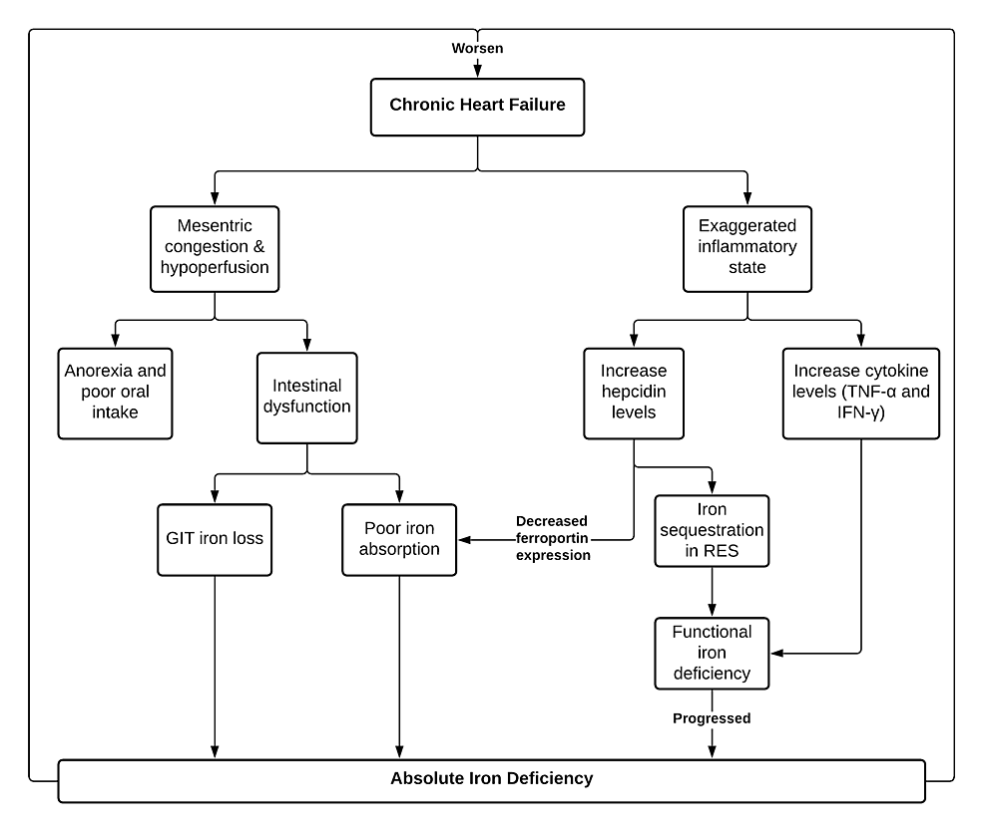
Flowchart of Interconnected Pathways Between Chronic Heart Failure and Iron Deficiency GIT: Gastrointestinal Tract, RES: Reticuloendothelial System, TNF-α: Tumor Necrosis Factor-α, IFN-γ: Interferon-γ

Dysfunctional cardiomyocyte's cellular metabolism relating to iron deficiency can potentially cause cardiomyocyte contractility dysfunction [46]. The decreased cardiomyocytes' myoglobin content, hence, reduced cellular antioxidant capacity, is one factor contributing to cardiac fibrosis and myocardial depression [37]. Long-standing iron deficiency positively correlates with myocardial fibrosis's progressivity, left ventricular systolic dysfunction, and the clinical course of heart failure [47]. The pathomechanisms of iron deficiency in heart failure patients are summarized in Figure 1.

Clinical implication of iron deficiency in chronic heart failure

Studies in the past decade have found that iron deficiency is important comorbidity often found in CHF [48]. However, proper evaluation and management of iron deficiency in CHF are frequently disregarded when not accompanied by anemia. Furthermore, iron deficiency without anemia symptoms is unspecific, causing it to be easily overlooked. Therefore, a systematic evaluation of various laboratory indicators is necessary to diagnose iron deficiency [49].

The prevalence of iron deficiency in CHF regardless of anemia is relatively high, around 30-50% [12,48,50]. A cohort of 1506 systolic heart failure patients reported that iron deficiency is more commonly found in patients with anemia (61.2%) while the prevalence is also high in patients without anemia (45.6%). Other predictors of iron deficiency in CHF include female gender, higher New York Heart Association (NYHA) functional classification, higher N-terminal pro-brain-type natriuretic peptide (NT-proBNP) level, and lower erythrocyte mean corpuscular volume [12]. Another study also showed that iron deficiency is more commonly found in patients with higher high-sensitivity C-reactive protein (hs-CRP) levels [48]. Meanwhile, there was no significant correlation between antiplatelet and anticoagulant use with the incidence of iron deficiency in CHF patients [43].

Iron deficiency is also thought to contribute to the progressivity of structural dysfunction and remodeling in CHF. In an experimental animal model of iron deficiency studied for 12 weeks, it was found that there were left ventricular dilatation, mitochondrial macro-structure abnormalities, sarcomere structure irregularities, and an increase in oxidative stress response, including an increase in mitochondrial C cytochrome and reactive nitrogen species (RNS) [51].

Several studies in the last few years reported the link between iron deficiency and worse clinical outcomes in CHF patients. Univariate and multivariate analysis in 546 systolic heart failure patients, ranging from mild to severe delineated iron deficiency as a strong independent predictor towards mortality and heart transplant [12,48]. Another study in 157 patients with CHF also supported the data finding iron deficiency to increase mortality rate, independent of anemia [43]. Furthermore, iron deficiency in CHF also contributes to poorer QoL. It impacts aerobic capacity, endurance, physical activity, and work performance [27,43]. It is also attributed to cognitive, behavioral, and emotional dysfunction [52]. Lastly, CHF patients with iron deficiency have been reported to have a tendency for psychological and emotional disorders [52].

Owing to the significant impact of CHF on mortality, morbidity, and QoL, the issue of iron deficiency should not be underestimated, and adequate therapy with proven clinical efficacy and safety needs to be known by all cardiologists. Thus, we summarized the relevant evidence from randomized clinical trials and systematic reviews/meta-analyses. Numerous studies have demonstrated the clinical benefit of intravenous iron administration in CHF patients. A randomized clinical, prospective, double-blind study by Toblli et al. reported that the intravenously administered iron sucrose (5 doses, 200 mg each week) lowers NT-proBNP and CRP levels significantly compared with placebo [53]. The subjects were 40 systolic heart failure patients (ejection fraction ≤35%) with anemia, iron deficiency (serum ferritin <100 μg/L and/or transferrin saturation <20%), and mild renal dysfunction. The duration of observation was six months. The iron sucrose group also exhibited a substantial improvement in their NYHA functional class, Minnesota Living with Heart Failure (MLHF) score, 6-minute walk test (6MWT) distance, and ejection fraction.

The Ferinject Assessment in Patients with Iron Deficiency and Chronic Heart Failure (FAIR-HF) study, a large scale clinical trial of intravenous iron administration in chronic heart failure study, compared intravenous FCM preparation administration from placebo in a prospective, randomized, double-blind, multicenter trial of 459 heart failure patients with the NYHA functional class of II-III [54]. Iron deficiency is defined as a ferritin level of <100 µg/L or ferritin level between 100-299 µg/L with transferrin saturation of <20%. Subjects consist of patients with or without anemia, with a hemoglobin level between 9.5 and 13.5 g/dL. The correction phase of 200 mg of intravenous FCM administration weekly occurs for eight to 12 weeks. Then it is followed by a maintenance phase of 200 mg intravenous FCM administration every four weeks. At the end of the 24-week monitoring, the group with intravenous iron had a significant increase in hemoglobin and serum ferritin levels. This increase in iron level is also accompanied by clinical improvement in heart failure patients, including improvement in symptoms, improvement in functional capacity (50% in the iron group and 28% in the placebo group; p<0.001), QoL in relation to personal health, and 6MWT distance [54]. This clinical improvement is found in patients with or without anemia, even though hemoglobin levels did not change after intravenous iron administration in the non-anemic sub-group.

Then, after four years of hiatus regarding iron therapy clinical trials in CHF patients, several studies started to emerge. The IRON-HF study in 2013 was a multicenter, randomized, double-blind, placebo-controlled trial that aimed to compare the effects of intravenous iron versus oral iron supplements in anemic heart failure patients [55]. The interventions given were intravenous iron sucrose 200 mg once a week for five weeks, oral ferrous sulfate 200 m, three times daily for eight weeks, or placebo. It was found that intravenous iron seems to be superior in improving the functional capacity of heart failure patients with an increment of 3.5 ml/kg/min in peak oxygen consumption in the intravenous iron group and no increment in peak oxygen consumption in the oral iron group.

Ferric Carboxymaltose (FCM) Evaluation on Performance in Patients with Iron Deficiency in Combination with Chronic Heart Failure (CONFIRM-HF) in 2015 was a multicenter, double-blind, double-blind, placebo-controlled trial that enrolled 304 symptomatic, iron-deficient HF patients to assess the potential long-term impact of intravenous iron therapy [56]. Patients were randomized to treatment with intravenous iron of FCM or placebo over one year. Treatment of symptomatic, iron-deficient HF patients with FCM over one year resulted in sustainable improvement in functional capacity, symptoms, and QoL as assessed by an improvement in NYHA class, patient's global assessment, QoL score, and fatigue score. In addition, treatment may also be associated with a risk reduction of hospitalization for worsening heart failure.

A study in CHF patients with chronic kidney disease and iron deficiency anemia as comorbids demonstrated the benefit of 200 mg/week intravenous iron administration for five weeks to improve cardiac structure and function, aside from clinical improvement [57]. By the end of the six months evaluation period, subjects with iron therapy displayed significant progress in heart failure symptoms, NYHA functional class, NT-pro-BNP level, hemoglobin level, transferrin saturation, and ferritin level. Iron therapy also improved on left ventricular function and structure, which were demonstrated by echocardiography parameter changes, including a decrease in left ventricular diastolic diameter (LVD), left ventricular systolic diameter (LVSd), left posterior ventricular wall (LVPW), and left ventricular ejection fraction. A decline in inflammatory status was assessed by CRP levels, reflecting the positive response towards iron therapy. Thus, this study supports the hypothesis that cardiac function improvement has a parallel relationship with iron status in CHF patients.

The Effect of FCM on Exercise Capacity in Patients with Iron Deficiency and Chronic Heart Failure (EFFECT-CHF) [58] study in 2017 was a prospective randomized controlled, multicenter, open-label trial with blinded endpoint evaluation. They aimed to examine the effect of treatment with intravenous FCM compared with standard care, on exercise capacity in patients with symptomatic CHF and iron deficiency in 172 patients with systolic heart failure (left ventricular ejection fraction ≤45%) and mild to moderate symptoms despite optimal heart failure medication. After 24 weeks of FCM administration, it was found that the peak oxygen consumption had significantly decreased in the control group compared with the FCM group. The patients' global assessment and NYHA functional class improved in the FCM group.

The European Society of Cardiology in 2016 recommended that iron status should be evaluated as part of the initial work-up of all newly diagnosed heart failure patients [4]. Ferritin and transferrin saturation are blood markers that can be used for the diagnosis of iron deficiency. Iron deficiency is treated based on a serum ferritin level of <100μg/L or 100-299μg/L when transferrin saturation is <20%. Ferritin and transferrin saturation testing should be performed simultaneously and evaluated together. Current treatment options to correct iron deficiency in the general population are intravenous or oral iron. In symptomatic patients with systolic HFrEF (LVEF >40%), iron deficiency is treated with intravenous FCM. Similarly, the American Heart Association in 2017 also recommended that in heart failure patients with NYHA class II and III, and iron deficiency (ferritin <100 ng/mL or 100 to 300 ng/mL if transferrin saturation is <20%), intravenous iron replacement might be reasonable to improve functional status and QoL [59].

The iron preparation recommended for CHF patients is in the form of intravenous iron [8]. The use of oral iron preparation has limitations such as limited effectivity, limited absorption, gastrointestinal side effects, and the potential for interaction with heart failure medications. In the functional iron deficiency setting, intravenous iron has better effectiveness than the oral counterpart [60]. The Ganzoni formula has been used to calculate the amount of iron deficiency and the total dose of iron supplementation needed [61]. Total iron dose (mg) = weight (kg) x [15 - actual hemoglobin (g/dL)] x 2.4 + 500.

Table 2 and Table 3 summarize other completed and ongoing clinical trials, respectively.

**Table 2:** Summary of Completed Clinical Trials HF = Heart Failure; HfrEF = Heart Failure Reduced Ejection Fraction; NYHA = New York Heart Association; LVEF = Left Ventricular Ejection Fraction; ADHF = Acute Decompensated Heart Failure; TSAT = Transferrin Saturation; Hb = Hemoglobin; 6MWT = 6 Minute Walk Test; PE = Primary Endpoint; SE = Secondary Endpoint; NT-proBNP = N Terminal- pro Brain Natriuretic Peptide; BNP = Brain Natriuretic Peptide; BMI = Body Mass Index; KCCQ = Kansas City Cardiomiopathy Questionnaire; QoL = Quality of Lif; PGA = Patient Global Assessment; RR = Respiratory Rate; ADP = Adenosine Diphosphate; VAS = Visual Analog Scale; VO2 = Rate of Oxygen; LVDd = Left Ventricular Diastolic Dysfunction; LVSd = Left Ventricular Systolic Dysfunction

Tittle	First Author	Type	Diagnosis	Operational Definition	Number of patients	Randomization group	Dosage	Duration	Outcome
Beneficial Effects of Long-Term Intravenous Iron Therapy with Ferric Carboxymaltose in Patients with Symptomatic Heart Failure and Iron Deficiency (CONFIRM-HF) [56]	Piotr Pinokowski, 2015	Multi-centre, double-blind, placebo-controlled trial	HF patients with LVEF≤45%, elevated natriuretic peptides, and iron deficiency	HF patients: 1) Stable ambulatory HF patients with (NYHA II) or (NYHA III), with LVEF ≤45%, and elevated NP Iron deficiency anemia: 1) Serum ferritin level <100 ng/mL or between 100 and 300 ng/mL if TSAT<20% 2) Hb <15 g/dL	304	152:152 (FCM vs Placebo)	Intravenous FCM 200 mg weekly	52 weeks	PE 1. Improved 6MWT distance significantly at week 24 SE 1. Improved NYHA class 2. Improved PGA 3. Improved QoL 4. Improved Fatigue Score
Changes in Echocardiographic Parameters in Iron Deficiency Patients with Heart Failure and Chronic Kidney Disease Treated with intravenous iron [57]	Jorge E Toblii, 2015	Double-blind, randomized, placebo-controlled study	HFrEF, CKD, and iron deficiency anemia	HFrEF : 1) LVEF < 35% 2) NYHA II-IV 3) receiving optimal treatment for HF CKD : CrCl < 90 mL/min iron-deficiency anaemia : 1) Hb < 12.5 (men) and Hb < 11.5 (female) 2) Serum ferritin < 100 ng/mL and/or TSAT < 20%	60	1:1 (IS vs Placebo)	IV iron sucrose (IS) treatment 200mg/200 mL weekly	25 weeks	1.↑ LVEF (p<0.01) 2. improved in NYHA functional class (p<0.01) 3. ↓ LVSd and LVDd (p<0.01) 4. not significantly change in LVPW (P=0.027) 5. not significantly change in IVS (P=0.099) 3. ↑ Hb, ferritin and TSAT (p<0.01) 4.↑ renal function (p<0.01) 5.↓ NT-pro-BNP (p<0.01) 6.↓ inflammatory marker (p<0.001) 7. ↓ Heart rate and BMI (p<0.01)
Effect of Ferric Carboxymaltose on Exercise Capacity in Patients With Chronic Heart Failure and Iron Deficiency [58]	Dirk J. van Veldhuisen, 2017	prospective randomized controlled, multicenter, open-label trial with blinded end-point evaluation	HFrEF and iron deficiency anemia	HFrEF : 1) LVEF ≤45% and had to be performed in ≤3 months of screening 2) LVEF≤45% and >3 months after stable β-blocker therapy or device implantation 3) BNP >100 pg/mL or NT-proBNP >400 pg/mL 4) NYHA class II–III Iron deficiency anemia : 1) serum ferritin = 0 - 300 ng/mL 2) TSAT <20%	172	1:1 (FCM vs Placebo)	Day 0 1) Hb ≤14 g/dL --> 1000 mg FCM (20 mL), whereas patients 2) Hb >14g/dL --> 500 mg FCM (10 mL). Week 6, 1) Hb <10 g/dL <70 kg --> second dose of 500 mg FCM, ≥70 kg --> second dose of 1000 mg FCM 2) Hb 10 - 14 g/dL --> second dose of 500 mg FCM, 3) Hb >14 g/dL --> no additional dose Week 12 if serum ferritin was <100 ng/mL or if ferritin was 100 to 300 ng/mL with TSAT <20% --> third dose of 500 mg FCM	24 weeks	PE : not significantly change in peak VO2 (P=0.23) SE : 1. NTproBNP (P=0.13) did not significantly change 2. Improved NYHA functional class (P<0.05) 3. ↑ Hb, ferritin and TSAT (P<0.05) 4. ↑ Patient global assessment (P<0.05)
Effect of Oral Iron Repletion on Exercise Capacity in Patients with Heart Failure With Reduced Ejection Fraction and Iron Deficiency [62]	Gregory D, 2017	double-blind, placebo-controlled randomized clinical trial	HFrEF and iron deficiency anemia	HFrEF : LVEF <40% Iron deficiency anemia 1) serum ferritin = 15 - 299 ng/mL 2) TSAT < 20%	225	111:114 (Oral iron vs Placebo)	Oral iron polysaccharide 150 mg twice daily	16 weeks	PE : not significantly change peak VO^2^ (P = 0.46) SE: not significant changes in 6-minute walk distance, NT-proBNP levels, and KCCQ score (P > 0.05)
Identifying responders to oral iron supplementation in heart failure with a reduced ejection fraction: a post-hoc analysis of the IRONOUT-HF trial [63]	Andrew P. Ambrosy, 2018	double-blind, placebo-controlled, randomized clinical trial	HFrEF and iron deficiency anemia	HFrEF : 1) LVEF ≤ 40% 2) NYHA functional class II–IV Iron deficiency 1) ferritin 15–100 ng/ml or ferritin 100–299 ng/ml 2) TSAT <20%	98	24 : 74 (Oral iron : Placebo)	oral iron polysaccharide 150 mg twice daily	16 weeks	PE: not significantly change in peak VO2 (P=0.74) and ventilatory anaerobic threshold (P=0.37) SE: not significantly change in NT-proBNP (P=0.77), clinical KCCQ summary score (P=0.60), and overall KCCQ summary score (p=0.55)
Single-dose intravenous iron in Southeast Asian heart failure patients (PRACTICE-ASIA-HF) [64]	Tee Joo Yeo, 2018	randomized placebo-controlled study	ADHF snd iron deficiency anemia	ADHF : regardless of ejection fraction Iron deficiency anemia: 1) serum ferritin<300 ng/mL 2) TSAT <20% 3) Hb ≤14 g/dL	50	1:1 (FCM vs Placebo)	IV FCM 1000 mg before discharge	12 weeks	PE : not significantly change in 6MWT distance (P = 0.956) SE: not significantly change in KCCQ (P = 0.670) and VAS (P = 0.386)
Effect of Iron Isomaltoside on Skeletal Muscle Energetics in Patients with Chronic Heart Failure and Iron Deficiency: The FERRIC-HF II Randomized Mechanistic Trial [65]	Geoffrey Charles-Edwards, 2019	randomized, double-blind, placebo-controlled, mechanistic trial	HFrEF and iron deficiency anemia	HFrEF: 1) NYHA class II with LVEF ≤40% within the preceding 6 months 2) NYHA class III and LVEF ≤45% within the preceding 6 months 3) use of optimal HF drugs for ≥4 weeks without dose changes for ≥2 weeks Iron deficiency anemia 1) Hb <12,0 g/L in females and < 13,0 g/L in males 2) Ferritin <100 ng/L or 100-300 ng/L 3) TSAT <20%	40	21 : 19 (IIM vs placebo)	Iron (III) isomaltoside 100mg iron/mL ampoules body weight (kg) x 2.4 x (15 - patients Hb[g/dL]) + 500 mg (for stores) 19 Doses of 0-10 mg/kg and 11-20 mg/kg were infused over 30 and 60 minutes respectively. Doses exceeding 20 mg/kg were split and given at 2 separate sittings 1 week apart	2 weeks	PE : ↑ skeletal muscle PCr t1/2 (P=0.006) SE : 1) ↑ADP, t1/2 (P=0.02), 1) ↑ ferritin (P<0.0001), 3) ↑TSAT(P=0.002) 4) ↑NYHA class ( P=0.04) 5) ↑resting RR (P=0.009) 6) ↑post-exercise Borg dyspnea score ( P=0.04) 7) not significantly change Hb (P=0.41)
Noninvasive Imaging Estimation of Myocardial Iron Repletion Following Administration of Intravenous Iron: The Myocardial-IRON Trial [66]	Julio Nunez, 2020	investigator-initiated, multicenter, double-blind, randomized clinical trial	HFrEF and iron deficiency anemia	HFeEF : 1) LVEF <50% 2) NYHA functional class II–III Iron deficiency anemia 1) serum ferritin <100 lg/L or 100–299 lg/L 2) TSAT <20% 3) Hb <15 g/dL	53	27:26 (FCM vs Placebo)	20-mL IV FCM (1000 mg of iron) administered over at least 15 minutes		PE: T2* and T1 mapping were significantly lower ( P=0.025; and P=0.001) (7 days) T2*mapping were significantly lower in 30 days (P=0.003) T1 mapping were not significantly changed in 30 days (P=0.577) SE: Improved in KCCQ (P<0.001) Improved in NYHA functional class (P<0.001) Not significantly lowering NT-proBNP Not significantly change in 6MWT Not significantly change in LVEF

**Table 3:** Summary of Ongoing Clinical Trials HF = Heart Failure; HfrEF = Heart Failure Reduced Ejection Fraction; AMI = Acute Myocardial Infarction; CV = Cardiovascular; IV = Intravenous; MRI = Magnetic Resonance Imaging; NYHA = New York Heart Association; LVEF = Left Ventricular Ejection Fraction; ADHF = Acute Decompensated Heart Failure; TSAT = Transferrin Saturation; Hb = Hemoglobin; 6MWT = 6 Minute Walk Test; PE = Primary Endpoint; SE = Secondary Endpoint; NT-proBNP = N Terminal- pro Brain Natriuretic Peptide; BNP = Brain Natriuretic Peptide; BMI = Body Mass Index; KCCQ = Kansas City Cardiomiopathy Questionnaire; QoL = Quality of Lif; PGA = Patient Global Assessment; RR = Respiratory Rate; ADP = Adenosine Diphosphate; VAS = Visual Analog Scale; VO2 = Rate of Oxygen; LVDd = Left Ventricular Diastolic Dysfunction; LVSd = Left Ventricular Systolic Dysfunction

No.	Study	Principal investigator	Study start date	Estimated study completion date	Study design	Subjects	Intervention	Control	Duration of observation/treatment	Primary Outcome	Secondary outcome
1	FAIR-HF2 [67] NCT03036462	Mahir Karaks, MD	February 7, 2017	December 2021	International, prospective, multi-entre, double-blind, parallel-group, randomized, controlled, interventional trial	HFrEF & Iron Deficiency	FCM 1000 mg IV, followed by an optional administration of 500-1000 mg within the first 4 weeks, followed by administration of 500 mg FCM at every 4 months, except when haemoglobin is > 16.0 g/dL or ferritin is > 800 µg/L	NaCl IV	12 month	Combined rate of recurrent cardiovascular hospitalizations and of cardiovascular death	1) Combined rate of recurrent hospitalizations for any reason and of cardiovascular death 2) Combined rate of recurrent hospitalizations for any reason and of cardiovascular death 3) Rate of recurrent cardiovascular hospitalizations 4) Rate of recurrent HF hospitalizations 5) Rate of recurrent hospitalizations of any kind 6) All-cause mortality 7) cardiovascular mortality 8) Changes in NYHA functional class 9) Changes in 6-minute walk-test 10) Changes in EQ-5D 11) Changes in Patient Global Assessment (PGA) of wellbeing 12) Changes in renal laboratory parameters 13) Changes in cardiovascular laboratory parameters 14) Changes in inflammatory laboratory parameters 15) Changes in metabolic laboratory parameters
2	HEART-FID [68] NCT03037931	Adrian F Hernandez, MD	March 15, 2017	June 2022	Double-blind, multicenter, prospective, randomized, placebo-controlled study	HFrEF & Iron Deficiency	FCM 2x15mg/kg IV (max individual dose: 750mg) 7 days apart, repeated every 6 months as indicated by iron indices	NaCl 15ml - 2 doses 7 days apart repeated every 6 months	12 month	1) Incidence of Death (Time Frame: 1 year ) 2) Incidence of hospitalization for heart failure [Time Frame: 1 year] 3) Change in 6MWT distance [ Time Frame: 6 months ]	NA
3	Affirm-AHF [69] NCT02937454	Piotr Ponikowski, MD	April 3, 2017	August 2020	Randomized, double-blind, placebo-controlled trial	HFrEF & Iron Deficiency	FCM IV	NaCl IV	52 weeks	HF hospitalizations and CV death up to 52 weeks after randomization	1) Recurrent CV hospitalizations and CV death 2) HF hospitalizations 3) CV mortality 4) The composite of HF hospitalizations or CV death 5) Days lost due to HF hospitalization or CV death
4	FAIR-HFpEF [70] NCT03074591	Wolfram Doehner, Prof	August 2017	July 2021	Randomized, placebo-controlled trial	HFpEF & iron deficiency	Ferric Carboxymaltose 50mg/ml IV 15ml	NaCl IV	52 weeks	The difference of 6-minute walking distance	1) Changes in PGA quality of life questionnaire 2) Changes in NYHA functional class 3) Changes in mortality and Heart failure-related hospitalization rates
5	PREFER-HF [71] NCT03833336	José Luis Morales Rull, MD, PhD	August 23, 2017	June 2020	Randomized, placebo-controlled trial	HFpEF & iron deficiency	1) Ferric carboxymaltose 500-1000 mg IV at 0,6,12,24 weeks 2) oral capsules of ferroglycine sulfate iron 2x100 mg until week 24 3) oral capsules of Sucrosomial iron 2x30 mg until week 24	Normal saline solution plus oral lactose capsules	24 weeks	Changes in 6-minute walking test distance	1) Changes in NYHA functional classification 2) Changes in Quality of Life assessed by Kansas City Cardiomyopathy Questionnaire 3) HF-related or other cardiovascular hospitalizations 4) all causes and cardiovascular mortality
6	Ferric Carboxymaltose to Improve Skeletal Muscle Metabolism in Heart Failure Patients With Functional Iron Deficiency [72] NCT03218384	Stuart Katz, MD	September 7, 2017	August 2020	Randomized, double-blind, interventional study	Symptomatic NYHA Class II-III heart failure >3 months & iron deficiency	1) Ferric Carboxymaltose 750 mg per 15 ml injectable solution	Normal saline	4 weeks	Post-exercise phosphocreatine recovery time measured non-invasively with 31P-magnetic resonance spectroscopy	1) Change in 6-minute walk test distance from the baseline to 4 weeks 2) Change in Kansas City Cardiomyopathy Questionnaire score from baseline to 4 weeks
7	IronEx [73] NCT03803111	Dr. Harm Wienbergen	September 2020	December 2021	Randomized, open-label trial	HFrEF NYHA class II-III & iron deficiency	1) Intravenous iron supplementation with Ferric carboxymaltose, subsequent (after 2 months) exercise training program 2) Exercise training program, subsequent (after 2 months) intravenous iron supplementation with Ferric carboxymaltose	-	4 months	Exercise capacity (Peak VO2) change from baseline to 4 months	1) Change in 6 Minute walking distance 2) Change in New York Heart Association class 3) Change in echocardiographic ejection fraction of left ventricular function 4) Combined endpoint cardiovascular hospitalizations and death after 2 and 4 months
8	Iron Deficiency in Heart Failure Patients [74] NCT03883854	Essam Nan Saleeb, MD	March 25, 2019	October 20, 2020	Observational, cross-sectional	HFrEF	-	-	-	percentage of iron deficiency in systolic heart failure patients	-
9	Carenfer IC [75] NCT03924258	-	May 15, 2019	June 30, 2019	Open-label diagnostic trial	HF regardless of LVEF	Complete blood iron status		-	prevalence of iron deficiency in patients with Heart Failure	-
10	IV Iron in Acute Decompensated Heart Failure [76] (FERRLECITR) NCT04063033	Erez Marcusohn MD	September 1, 2019	August 31, 2022	Randomized, assessor-blinded, interventional study	HF regardless of LVEF & iron deficiency	Sodium Ferric Gluconate Complex 125 mg IV /day for 3-5 days	standard treatment for heart failure without IV Iron	1 year	Functional Capacity change from baseline, 12 weeks, and 24 weeks	1) Change in NYHA from baseline to 12 and 24 weeks 2) Incidence of all-cause mortality up to 1-year follow-up 3) Incidence of hospitalizations due to heart failure up to 1-year follow-up
11	iCHF-2 [77] NCT03991000	Mahir Karakas, MD, MBA	February 28, 2019	June 2023	Randomized, quadruple-blinded, interventional study	1) AMI & iron deficiency 2) AF & iron deficiency 3) HFrEF & iron deficiency	Bolus administration of ferric carboxymaltose (1000 mg) followed by an optional administration of 500-1000 mg within the first 4 weeks (up to a total of 2000 mg which is in-label) followed by administration of 500 mg at months 4 and 8, except when haemoglobin is > 16.0 g/dL or ferritin is > 600 µg/L	i.v. NaCl according to the dosing rules for intravenous iron.	1 year	1) Change from baseline to week 16 in left-ventricular ejection fraction as determined by cardiac-MRI 2) Delta between treatment groups in the burden of atrial fibrillation from day 90 to 365 as assessed by a routinely implanted event recorder 3) Change from baseline to week 16 in left-ventricular ejection fraction as determined by cardiac-MRI	-

The long-term effectivity and safety profile of intravenous iron therapy in CHF patients with iron deficiency still need to be confirmed by future large-scale studies. Some clinical questions regarding iron therapy yet to be answered by future studies include the benefit of oral iron therapy compared to intravenous therapy, the benefit and long-term effect of iron therapy in the clinical course of heart failure [56], the use of laboratory parameters as therapeutic response assessment, and the outcome in studies with subjects on a larger scale with a more heterogeneous spread.

## Conclusions

Iron deficiency is prevalent in CHF, but it is often overlooked, especially without existing anemia. This comorbid might contribute to a worse clinical outcome in patients. Numerous studies have reported the clinical benefit of iron supplementation, particularly in intravenous preparation, in HF patients with regards to symptoms, functional capacity, and QoL improvement. Therefore, the current guidelines recommend routine screening of iron status in all newly diagnosed heart failure patients. Eventually, intravenous iron replacement is recommended for symptomatic HF patients with iron deficiency, irrespective of anemia.
